# Rapid Adaptive Evolution under Combination Therapy in *Klebsiella pneumoniae*

**DOI:** 10.1021/acsinfecdis.5c00934

**Published:** 2026-02-10

**Authors:** Camila Maurmann de Souza, Amy Lee, Osmel Fleitas Martínez, Kevin Ning, Mylena Cardoso da Costa, Mariana Rocha Maximiano, Gabriel Cidade Feitosa, Yasmim Neiva, Marcelo Campos, Marcelo Ramada, Sérgio Alencar, Robert E. W. Hancock, Octávio Luiz Franco

**Affiliations:** † Centro de Análises Proteômicas e Bioquímicas, Programa de Pós-Graduação Em Ciências Genômicas e Biotecnologia, Universidade Católica de Brasília, Brasília 71966-700, Brazil; ‡ S-Inova Biotech, Programa de Pós-Graduação Em Biotecnologia, 186072 Universidade Católica Dom Bosco, Campo Grande 79117-900, Brazil; § Department of Molecular Biology and Biochemistry, 1763 Simon Fraser University, South Sciences Building 7107, 8888 University Drive, Burnaby V5A 1S6, British Columbia, Canada; ∥ Integrative Plant Research Laboratory, Programa de Pós-Graduação Em Biologia Vegetal, 67826 Universidade Federal de Mato Grosso, Cuiaba 78060-900, Brazil; ⊥ Graduate Program in Gerontology, 28106 Catholic University of Brasilia, Brasilia 71966-700, Brazil; # Centre for Microbial Diseases and Immunity Research, 8166 University of British Columbia, 2259 Lower Mall Research Station, Vancouver V6T 1Z4, British Columbia, Canada

**Keywords:** *K. pneumoniae*, infectious disease, genomics, antibiotic resistance, bacteria, evolution

## Abstract

*Klebsiella pneumoniae* poses a substantial health concern worldwide, with high mortality often associated with its elevated resistance levels. Combination antibiotic therapies have emerged as a viable strategy for addressing infections caused by these highly resistant pathogens, yet the evolutionary routes to resistance under such regimens remain poorly understood. Here, we investigated how resistance can emerge during exposure to combination therapy by conducting an in vitro evolutionary experiment with a clinical *K. pneumoniae* KPC-producing isolate (KP03, Brazil), which was initially susceptible to both amikacin and polymyxin B (AmkPol). While the combination therapy displayed an additive effect in vitro, subinhibitory exposure rapidly drove resistance. In three independent lineages, bacteria tolerated concentrations nearly 10-fold (polymyxin B) and 5-fold (amikacin) above EuCAST breakpoints, with MICs reaching 128–256 μg·mL^–1^ after 45 passages. These high resistance levels persisted for at least 10 days without selective pressure, although resistance lineages exhibited measurable fitness costs. Whole-genome sequencing revealed diverse mutations affecting *lapB*, *phoP*, *rho*, *smbA*, *mlaA*, and *asmA*, while transcriptomics analysis showed upregulation of the *arn* operon and the *aphA* alongside with downregulation of envelope- and efflux-associated genes. Cross-resistance was also observed against colistin and certain antimicrobial peptides, raising concern for treatment options beyond the AmkPol combination. Although combination therapy represents an important treatment strategy, our findings demonstrate that *K. penumoniae* can rapidly evolve stable, high-level resistance under combination therapy, highlighting the need for a deeper understanding of how such regimens influence resistance development and the continued need to develop novel antibiotics strategies.

## Graphical abstract

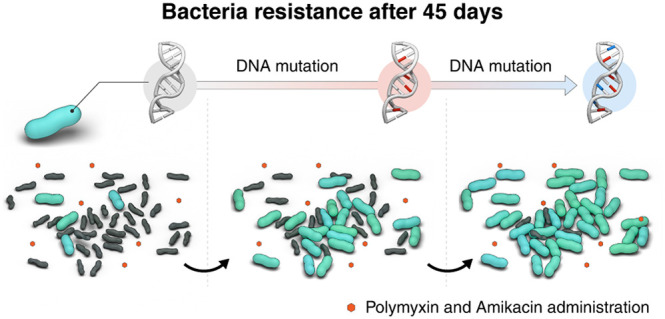

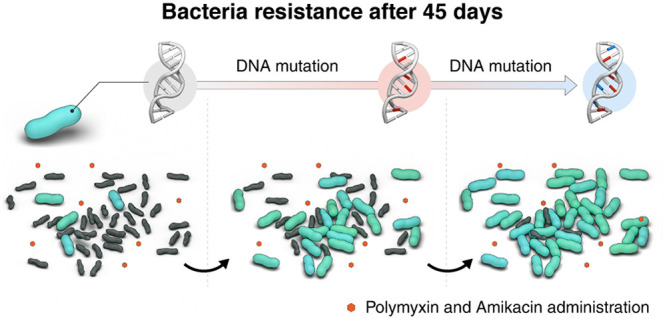

The rising mortality associated with antimicrobial resistance has become a major global health concern, with an estimated 1.27 million deaths reported in 2019 alone.^1^ Among the pathogens of critical priority, *K. pneumoniae* stands out due to its extensive repertoire of resistance mechanisms, the worldwide dissemination of high-risk clones, and mortality rates that can reach up to 50%.^2,3^ Given the limited availability of effective therapies and the ability of *K. pneumoniae* to cause infections that are difficult to treat, combination therapy has emerged as a commonly employed strategy in recent years.^4^ This approach, defined as the simultaneous use of two or more antimicrobial agents, seeks to enhance bactericidal activity, improve efficacy against resistant strains, and suppress or at least delay the emergence of resistance.^5,6^

Numerous antimicrobial combinations have been explored to improve the treatment of *K. pneumoniae*, representing a key strategy given the limitations of monotherapy.^7^ Among the combinations investigated, the combination of amikacin and polymyxin B has shown favorable in vitro activity and improved clinical outcomes in hospitalized patients.^8,9^ Polymyxins are commonly used in combination therapies against Gram-negative pathogens, including *K. pneumoniae*, to overcome antimicrobial resistance.^10–12^ Polymyxin B, a cyclic lipopeptide differing from colistin by a single amino acid, primarily disrupts the outer membrane by binding lipopolysaccharides and displacing divalent cations, with additional effects involving respiratory chain inhibition and reactive oxygen species generation.^13,14^ Amikacin, in contrast, is an aminoglycoside that inhibits protein synthesis by binding to the 30 S ribosomal subunit.^15^

Despite their potential, the mechanisms of action underlying antimicrobial combinations and their role in resistance emergence remain poorly understood.^16^ In vitro results often do not translate to clinical outcomes, and patient responses vary with therapeutic failures still reported. While combination therapy may benefit severe cases, monotherapy can sometimes be less risky by reducing the likelihood of superinfections. Overall, data are insufficient to fully evaluate the advantages of combination therapy versus the potential for promoting resistance.^17^ A more detailed understanding of the selective pressures imposed by combination therapy is critical to anticipate future challenges with multidrug-resistant pathogens and to inform the development of new antimicrobial agents.^1,3,4^

Experimental evolution studies provide valuable frameworks for evaluating bacterial adaptive trajectories under antimicrobial pressure, even before resistance becomes detectable in clinical settings.^18^ In parallel, omics approaches, including genomics and transcriptomics, have proven essential for identifying and characterizing resistance mechanisms.^19,20^

In this study, we aimed to investigate how resistance can emerge during combination therapy. For this purpose, we performed in vitro evolution experiments exposing a carbapenemase-producing *K. pneumoniae* clinical isolate (KPC) to subinhibitory concentrations of two distinct antibiotics with distinct mechanisms of action including amikacin and polymyxin B. Resistance dynamics were investigated using a multifaceted approach, including evaluation of fitness costs and stability, assessment of cross-resistance, and genomic and transcriptomic analyses to identify acquired resistance determinants and their implications for both current and emerging therapeutic strategies.

## Results

A high level of resistance against combinatory therapy can be achieved after subinhibitory concentration exposure.

The clinical isolate KP03 was obtained from a Brazilian hospital and initially was susceptible to both amikacin (MIC = 4 μg·mL^–1^) and polymyxin B (MIC = 2 μg·mL^–1^). When tested in vitro, the combination of these two antimicrobials exhibited an additive effect against KP03, reinforcing the potential of combination antibiotics as a therapeutic strategy (Figure 1A; Table S1). However, when subjected to subinhibitory concentrations of the AmkPol combination, the parental strain rapidly acquired resistance. Notably, in an experiment with 10 independent KP03 cultures, resistance emerged in nine lineages, which were able to survive exposure to 19.2 μg·mL^–1^ polymyxin combined with 38.4 μg·mL^–1^ amikacin (Table S2). These concentrations represent nearly 10-fold and 5-fold increases over the EUCAST clinical breakpoints for polymyxin (2 μg·mL^–1^) and amikacin (8 μg·mL^–1^), respectively, highlighting the extraordinary capacity of KP03 to adapt under selective pressure. Moreover, three lineages (E1, E2, and E3) not only survived prolonged treatment but remained viable until the 45th passage, tolerating concentrations as high as 43.2 μg·mL^–1^ of polymyxin and 86.5 μg·mL^–1^ of amikacin. These resistant lineages exhibited MIC values ranging from 128 to 256 μg·mL^–1^ for both antimicrobials (Figure 1B; Table S3), corresponding to an increase of 32–64-fold for amikacin and 64–128-fold for polymyxin B. Together, these findings demonstrate how fast *K. pneumoniae* can develop resistance under combination therapy but also the magnitude of the resistance that can arise, raising concerns about the long-term effectiveness of such therapeutic strategy.

**Figure 1:**
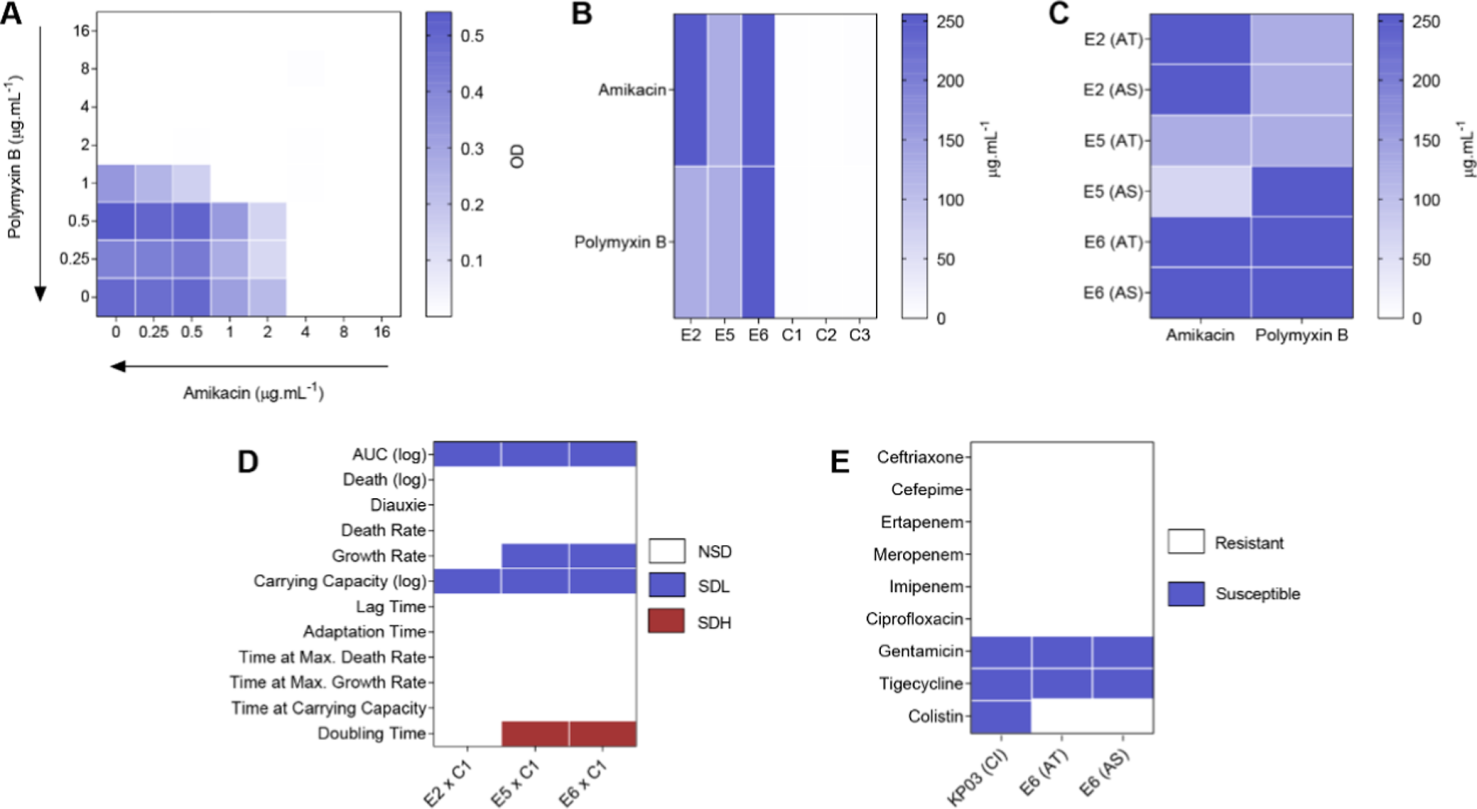
*Klebsiella pneumoniae* parameters’ analysis. (A) Checkerboard results of polymyxin B and amikacin combination against clinically isolated *K. pneumoniae* 03 (KP03). (B) Minimum Inhibitory Concentration (MIC) of amikacin and polymyxin B against *K. pneumoniae* lineages after in vitro evolutionary trajectory. (C) Resistance stability of *K. pneumoniae* lineages after 10 days without amikacin and polymyxin B combination challenge. (D) Comparison of growth parameters between the resistant lineages (E2, E5, E6) against the control lineage (C1); NDS: not significantly different, SDL: significantly different with lower mean, SDH: significantly different with higher mean. (E) Cross-resistance analysis between parental lineage KP03 and E6 resistant lineage after in vitro trajectory and stability assay; CI: clinical isolate, AT: after trajectory, AS: after stability.

### Development of Stable Resistance

The amikacin and polymyxin B resistance developed by the resistant lineages during in vitro evolution proved to be stable, persisting even after 10 consecutive days of growth in the absence of antibiotic pressure. MIC values during this period remained elevated, ranging from 64 to 256 μg·mL^–1^ (Figure 1C, Table S4). Although a modest reduction in amikacin resistance was observed in lineage E5, where the MIC decreased from 128 to 64 μg·mL^–1^, this shift corresponds to concentrations far above the EUCAST clinical breakpoints. Importantly, all other lineages consistently retained high-level resistance, underscoring the durability and adaptive mechanisms selected under combination therapy. These findings indicate that once established, resistance in *K. pneumoniae* is not only rapid but also resilient, persisting without continuous selective pressure.

### Resistance to Combination Can Be Associated with Fitness Costs

When resistant lineages (E2, E5, and E6) were compared with the control strain (C1), marked differences emerged in key growth parameters, including the area under the curve (AUC), growth rate, the carrying capacity, and doubling time (Figure 1D, Tables S5 to S7). All three resistant strains displayed reduced values in both AUC and carrying capacity relative to the control, indicating an overall impairment in their ability to sustain growth. Moreover, lineages E5 and E6 exhibited an ever more pronounced decline in fitness, with significantly lower growth rates and extended doubling times. These results suggest that while resistance to the AmkPol combination can provide survival advantages under selective pressure, this comes at the expense of reduced growth, with certain lineages experiencing a significant fitness burden.

### Different Genetic Responses Are Related to Combined Therapy Resistance

To uncover the genetic basis of resistance following the experimental evolution experiment, we sequenced the parental strain (KP03), control lineages (C1, C2, C3), and resistant lineages (E2, E5, E6). Our analysis revealed a diverse set of mutations, deletions, and substitutions unique to each resistant lineage (Table 1), indicating that *K. pneumoniae* can acquire resistance to AmkPol through multiple evolutionary routes. In the E2 lineage, we identified a one-base pair deletion in the *mlaA* gene alongside a notable mutation in *rho*. By contrast, the E5 lineage accumulated multiple alterations, including substitutions in the *phoP* a T → G substitution in *lapB* and a deletion in the *smbA* (Table 1), pointing to complex remodeling of envelope integrity and stress response pathways. Lineage E6 displayed a broader deletion spanning multiple genes, including *asmA*, together with a C → T substitution in an intergenic region likely influencing *aphA* expression. Interestingly, the same intergenic mutation was also observed in E2, while E5 harbored a distinct directly impacts *aphA*.

**Table 1:** List of the Primary Mutations Found in Resistant Lineages (E2, E5, and E6) That Were Not Found in the Controls (C1, C2, and C3)^a^

position	mutation	E2	E5	E6	annotation	gene	description
80,533	T → A		X		I63F (ATC → TTC)	*phoP*_1	virulence transcriptional regulatory protein PhoP
27,041	Δ1 bp	X			coding (10,588/12,279 nt)	*smc*_1	chromosome partition protein Smc
199,440	A → G	X			D127D (GAT → GAC)	*mqo*_1	malate:quinone oxidoreductase
80,012	T → G		X		V43G (GTT → GGT)	*lapB*	lipopolysaccharide assembly protein B
63,193	C → T	X			A404T (GCA → ACA)	*rho*	transcription termination factor Rho
35,675	Δ2 bp		X		coding (321–322/1221 nt)	*sbmA*	peptide antibiotic transporter SbmA
1	Δ35,478 bp			X		*xylB*_4–KP03_04907	27 genes:*xylB_4*, *csbX_2*, *hxpB_2*, *gci*, *ddpA_2*, *ddpB*, *ddpC_3*, *oppD_2*, *oppF_3*, *yegS*, *rlhA_2*, *baeR*, *baeS*, *mdtD*, *mdtC*, *mdtB*, *mdtA_3, dctA_2*, *KP03_04899*, *KP03_04900*, *phoP_2*, *sasA_2*, *dnaK_2*, *alkA*, *udk*, *dcd*, *asmA*
22,449	Δ1 bp	X			coding (552/762 nt)	*mlaA*	intermembrane phospholipid transport system lipoprotein MlaA
6384	T → G			X	D567A (GAT → GCT)	KP03_	putative tyrosine-protein kinase in cps region
						05280	
8158	G → A	X			intergenic (−103/–96)	*mobA_2*/KP03_	mobilization protein A/hypothetical protein
						05302	
8166	G → A		X		intergenic (−111/–88)	*mobA_2*/KP03_	mobilization protein A/hypothetical protein
						05302	
8169	Δ1 bp			X	intergenic (−114/–85)	*mobA_2*/KP03_	mobilization protein A/hypothetical protein
						05302	
9050	C → T	X		X	intergenic (+512/–81)	KP03_	hypothetical protein/Aminoglycoside 3′-phosphotransferase
						05302/*aphA_2*	
9134	G → A		X		E2K (GAA → AAA)	*aphA_2*	aminoglycoside 3′-phosphotransferase
1	Δ3511 bp	X		X		KP03_	KP03_05398, *galF*
						05398–*galF*	

a Triangles identify deletions, arrows show substitutions, hyphens represent unknown data, and “X” marks the mutated lineage.

### The Transcriptional Landscape of *K. pneumoniae* Is Reshaped by AmkPol Exposure

To characterize the immediate bacterial response to the AmkPol combination, we performed RNA-seq analysis 1 h after antibiotic exposure, allowing us to capture the early transcriptional dynamics of lineage E6 in comparison with that of control strain C1. This approach identified 157 differentially expressed genes (DEGs), comprising 65 downregulated and 92 upregulated genes. Our analysis focused on resistome-associated genes that are likely to contribute to the adaptive response to the AmkPol combination. The primary genes of interest are displayed in Figure 2. Notably, several genes involved in envelope stability and efflux regulation, such as *galF*, *asmA*, *baeR*, *baeS*, *mdtA*, *mdtB*, *mdtC*, *mdtD* and *baeS*, were strongly downregulated. Many of these loci also carried deletions identified in the genomic analysis, and their lack of expression further reinforces their loss of function under antibiotic exposure.

**Figure 2:**
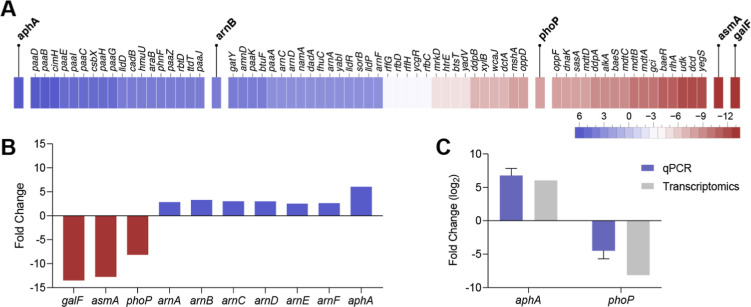
Putative resistance mechanisms to combining polymyxin B and amikacin in *K. pneumoniae*. (A) Heatmap showing the central genes differentially expressed in *K. pneumoniae* lineage E6 resistant to polymyxin B and amikacin when challenged with both antibiotics compared to a *K. pneumoniae* control lineage susceptible to the combination of antibiotics, with a log_2_ fold change ≥2 and ≤ −2. (B) Essential resistance genes exhibited significant upregulation or downregulation in the resistant strains (E6) compared to the control group (C1). (C) Differential expression analysis comparison of the genes *aphA* and *phoP* between resistant E6 and control C1. The log2 fold change obtained in transcriptomic analysis by comparing the same lineages was added to the graph below.

Conversely, we observed a pronounced upregulation in the *arn* operon (*arnA*, *arnB*, *arnC*, *arnD*, *arnF*), consistent with its known role in LPS modification and polymyxin resistance.^29^ In parallel, *aphA*, an aminoglycoside-modifying enzyme, was significantly upregulated, aligning with the genomic substitutions previously detected. To further validate these findings, we performed qPCR on *aphA* and *phoP*, confirming the upregulation of *aphA* and downregulation of *phoP*, consistent with the RNA sequencing data (Figure 2).

Together, these results reveal that *K. pneumoniae* resistance to AmkPol is underpinned by a coordinated transcriptional reprogramming that couples the activation of protective pathways, such as LPS remodeling and aminoglycoside modifications, with the silencing or disruption of genes linked to envelope maintenance and stress sensing.

### Cross-Evaluation of Resistance to Multiple Antimicrobial Compounds

To examine the potential for cross-resistance or collateral sensitivity, a comparative analysis was conducted between parental KP03 and the most resistant lineage E6 after the evolutionary process. A comparative analysis was also made against E6 after stability assays (10 passages without antibiotics). The MIC results (Figure 1) showed that no collateral sensitivity was obtained for the tested antimicrobial after the evolutionary trajectory, and this result remained after the stability assay.

Cross-resistance to colistin was observed with an MIC increased to 256 μg·mL^–1^. A comparable outcome was seen in the stability study with a value of 128 μg·mL^–1^. The MIC result suggests that the cross-resistance may also be stable. Likewise, both BotrAMP14 and mastoparan I5R8 demonstrated a MIC greater than 32 μM, suggesting the presence of cross-resistance. However, stability results show that cross-resistance against [I^5^,R^8^] mastoparan remains stable, while resistance against BotrAMP14 does not (8 μM). All of the other peptides tested had similar MIC results when comparing the parental strain and E6. PaDBS1R1 showed a modest 2-fold increase in MIC. However, this change likely does not reflect true cross-resistance, as a 2-fold variation can fall within the inherent sensitivity limits of the microdilution assay, Table 2.

**Table 2:** Antimicrobial Peptides’ Cross-Resistance Analysis between Parental *Klebsiella pneumoniae* KP03- and *Klebsiella pneumoniae* E6-Resistant Lineage after the In Vitro Trajectory and Stability Assay^a^

peptides	origem	MIC μM/(μg·mL^–1^)		
		clinical isolates	after in vitro evolution	after stability assay
		(KP03)	(E6)	(E6)
LL-37	*Homo sapiens*	32 (143.7)	>32 (>143.7)	>32 (>143.7)
BotrAMP 14	*Bothrops atrox*	4 (7.83)	>32 (>62.64)	8 (15.66)
CrotrAMP 14	*Crotalus durissus terrificus*	>32 (>58.25)	>32 (>58.25)	>32 (58.25)
L-mastoparan	*Vespula lewisii*	>32 (>47.32)	>32 (>47.32)	>32 (47.32)
[I^5^,R^8^] mastoparan	*Vespula lewisii*	4 (6.42)	>32 (>51.50)	32 (51.50)
PaDBS1R1	*Pyrobaculum aerophilum*	4 (8.43)	8 (16.86)	8 (16.86)

a LL-37 ^30^ and L-mastoparan^31^ are natural peptides, respectively, from humans and the venom of several wasp species. BotrAmp 14,^32^ CrotrAMP14,^32^ and [I^5^,R^8^] mastoparan^33^ are physicochemical design peptides, respectively, from the cathelicidins batroxicidin, crotalicidin, and from L-mastoparan. PaDBS1R1 is a template design peptide from an archaeon.^34^

## Discussion

In this study, we employed an experimental evolution framework, exposing cultures to progressively increasing subinhibitory concentrations of antibiotics to investigate the emergence of resistance during combination therapy. This evolutionary design mirrors clinical scenarios where antimicrobial levels remain below therapeutic thresholds due to factors such as poor tissue penetration, instability, or rapid clearance. Using this model, *K. pneumoniae* KPC producers were subjected to subinhibitory concentrations of an AmkPol combination.

Our findings show that although combination therapy remains an important strategy against bacterial infections, its use can also select for lineages with high-level resistance (Figure 1B). Thus, while often considered a resistance-suppressive approach, combination therapy may also act as a selective pressure that promotes resistance if not carefully managed. Documented treatment failures with antibiotic combinations against Gram-negative pathogens, together with the emergence of highly resistant “superbugs,” highlight the need for cautious and evidence-based implementation.^17,35^

We also observed that resistance development to AmkPol could persist for at least 10 days without antimicrobial pressure (Figure 1C). Although some studies indicate resistance loss in a nonselective medium^36,37^ our study suggests that resistant mutants maintained resistance, which could be challenging in hospital settings. In addition, we also examined the fitness cost as an indicator of growth and possible spread differences between susceptible and resistant strains. The development of resistance to AmkPol has associated a fitness cost,^38,39^ making the growth processes slower in the resistant lineages than in the control. However, differences were observed between E2, E5, and E6, suggesting that distinct mutations could confer different levels of fitness burden. Interestingly, although the development of AmkPol resistance involved a fitness cost, the resistance remained stable for at least 10 days, as mentioned above, suggesting either the involvement of compensatory mechanisms or that 10 days is insufficient time to observe reversion from the resistant to the susceptible phenotype.

Different mutations emerged in each resistant lineage along the evolutionary trajectory (Table 1), suggesting that resistance to this combination can be achieved through multiple pathways. Some mutations known to contribute to polymyxin resistance were found, including (1) in lineage E2, a deletion in *mlaA* that is responsible for lipid asymmetry maintenance in the outer membrane (OM) and has been associated with colistin resistance in *A. baumannii*, (2) a substitution in *phoP* detected in lineage E5 that could constitutively activate the PhoPQ two-component polymyxin resistance regulator,^40^ and (3) a substitution in *lapB*, in lineage E5, causing a V43G amino acid modification that has been linked with an increase in lipopolysaccharide (LPS) production that was associated with colistin resistance in *K. pneumoniae*.^41^ We also observed a substitution mutation in *rho* in lineage E2, and mutations in this gene also appeared in colistin-resistant strains after in vitro evolution in polymyxin.^42^ Moreover, *rho* overexpression has been associated with reactive oxygen species (ROS) resistance, which could be an essential mechanism against polymyxin B and amikacin, given that both antibiotics can promote ROS production.^14,43,44^ Conversely, all resistant lineages exhibited mutations in *aphA* or an intergenic region adjacent to this gene. The *aphA* gene encodes an aminoglycoside 3′ phosphotransferase that can mediate amikacin resistance.^45^ Despite our parental strain carrying this gene, it was susceptible to amikacin, and high levels of resistance were observed in the resistant strains. A plausible explanation for the difference in sensitivity to amikacin between the parental and resistant lineages, even though they all carried the *aphA* gene, could be a difference in the expression level of *aphA*. In this regard, under AmkPol-induced stress, the resistant E6 lineage upregulated the expression of *aphA* compared with the parental strain, which could explain the observed difference in sensitivity to amikacin and also leads us to hypothesize that the identified mutation in the intergenic region near this gene could influence the expression levels of the gene.

Finally, we meticulously characterized the initial transcriptional response to AmkPol through transcriptomics analysis in the E6 lineage, which exhibited the highest resistance to AmkPol. Previous studies with polymyxin and amikacin have demonstrated that early time points, particularly within the first hour of exposure, are marked by a high number of differentially expressed genes (DEGs) and pronounced metabolic alteration.^46–48^ This early phase of bacterial response remains relatively underexplored but is thought to reflect a rapid, innate-like defense strategy. Such an immediate response may serve to limit antibiotic-induced damage while the bacterial cell initiates a more specialized and coordinated defense mechanism.^47^

In our study, several of the DEGs were genes associated with the resistome of *K. pneumoniae* in response to antibiotics (Figure 2). As mentioned above, there was a significant upregulation of *aphA*, which would likely promote high levels of amikacin resistance. Moreover, the downregulation of *asmA* could be related to polymyxin resistance since diminished levels of glycerophospholipids may potentially lead to OM vesicle production enriched with LPS in order to maintain homeostasis.^46^ The production of OM vesicles has previously been related to deletion of other *asmA*-like genes. Furthermore, membrane vesicles are known for their protective mechanism against polymyxin B, probably through the adsorption of these molecules.^47,48^ Moreover, the upregulation observed in the *arn* operon would likely lead to lipid A modification (arabinosaminylation) of OM LPS (Figure 2) and could also lower AmkPol self-promoted uptake.^40^ These mechanisms potentially explain the acquisition of resistance to polymyxin B, combined with amikacin (Figure 3).

**Figure 3:**
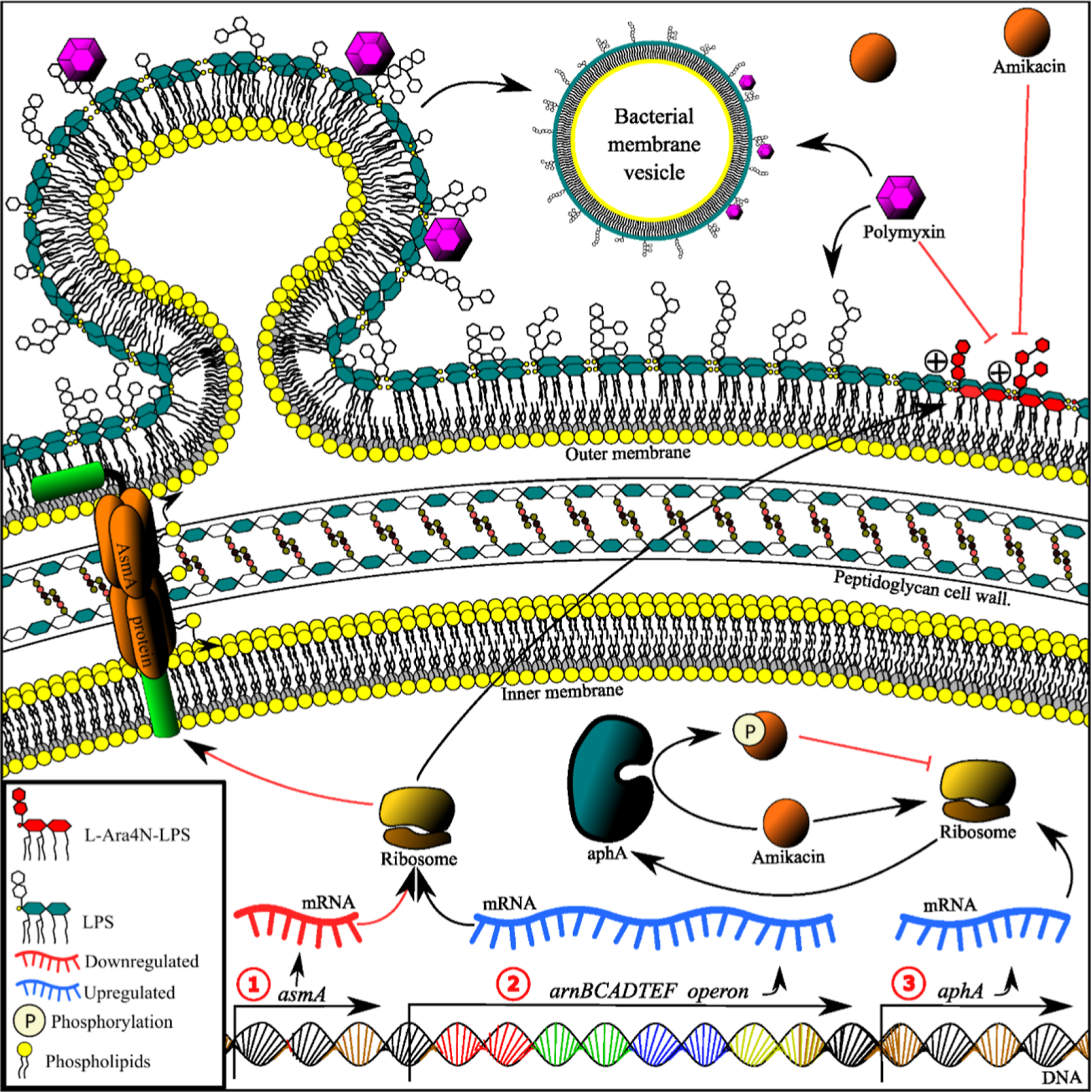
Putative resistance mechanisms when combining polymyxin B and amikacin in *K. pneumoniae*. (1) Downregulation of *asmA* could lead to asymmetry in the outer membrane, inducing membrane vesicle production, which could decrease polymyxin B entrance by trapping polymyxin B molecules binding to the LPS of these membrane vesicles instead of the outer membrane. (2) Activating the *arnBCADTEF* operon would lead to LPS modification by adding 4-aminoarabinose to lipid A, reducing the self-promoted uptake of polymyxin B and amikacin. (3) Upregulation of *aphA* that can catalyze amikacin O-phosphorylation, inhibiting its activity.

After characterizing resistance development and its underlying mechanisms, we sought to further evaluate how these changes could impact other treatment options. To this end, we analyzed the cross-resistance profile of the resistant lineage (E6) in comparison with the parental strain (KP03), assessing whether susceptibility patterns had shifted not only for conventional antibiotics but also for AMPs, which are emerging as promising therapeutic alternatives. Our results show that the KP03 resistance profile to antibiotics was mainly maintained in E6. Cross-resistance was observed only for colistin, which was expected since polymyxin B and colistin are both from the same class of antimicrobial agents and both act on the same bacterial target. Resistance factors identified in this study, such as *phoP* and the *arn* operon, are involved in resistance to both antimicrobial agents.^29^

Interestingly, the resistant lineage E6 displayed cross-resistance to some of the assayed AMPs, yet remained susceptible to the broad-spectrum membrane-active peptide PaDBS1R1. This suggests that cross-resistance is displayed for certain AMPs but not others. This opens the door to exploring AMPs exhaustively as an alternative therapy for strains that are resistant to the AmkPol combination.

## Conclusion

Although combination therapy represents an important therapeutic strategy against highly resistant bacterial infections, our findings suggest that under sub-MIC conditions combination therapy may not prevent and could even promote the development of resistance in certain *K. pneumoniae* lineages. Our data show that LPS modification diminished self-promoted uptake, and aminoglycoside modification can be promoted by subinhibitory concentrations of polymyxin B and amikacin combination. Moreover, resistance to this combination exerted differential effects on antimicrobial peptide (AMP) susceptibility, resulting in stable cross-resistance to peptides such as L-mastoparan and [I5,R8]-mastoparan, transient resistance that was lost following removal of selective pressure in BotAMP14, and no detectable cross-resistance to PaDBR1S1. These results highlight the importance of a more comprehensive analysis of how combination therapy influences resistance acquisition, as the emergence of highly resistant strains could pose a significant threat to human health. Additional studies should be conducted to gather more comprehensive insights into the mechanisms underlying the development of antimicrobial resistance and to explore novel therapeutic approaches.

## Methods

### Bacterial Strains

A clinical isolate of *K. pneumoniae* (KP03), a KPC-producing strain recovered from a hospital in Brazil, was used as the parental strain. From this isolate, we obtained lineages adapted to the Mueller–Hinton (MH) medium that remained sensitive to the amikacin–polymyxin B combination (C1, C2, and C3), as well as lineages that acquired resistance to the same combination (E2, E5, and E6). Control lineages were generated after 45 serial passages in MH medium alone, whereas resistant lineages were derived after 45 serial passages in MH medium supplemented with the antibiotic combination.

### In vitro Evolution of Antibiotics Combination Therapy Resistance

This study investigated the in vitro evolution of resistance to a combination of two antibiotics commonly used in tandem against *K. pneumoniae*: amikacin and polymyxin B. While these agents are no longer widely employed, polymyxin remains one of the last-resort treatments, and amikacin, with a completely distinct mechanism of action, provides a relevant model to study combination effects. Amikacin sulfate (A0365900) and polymyxin B sulfate (1547007) were purchased from Sigma-Aldrich.

Over 45 days, KP03 cultures were exposed to increasing concentrations of the antibiotic combination. Initial cultures were grown to ∼1 × 10^8^ CFU·mL^–1^, diluted to ∼5 × 10^5^ CFU·mL^–1^ in 96-well plates, and incubated for 24 h at 37 °C with agitation at 80 rpm. Cultures were then serially propagated in fresh Mueller–Hinton (MH) medium with or without the antibiotic combination to generate resistant and control lineages across 45 sequential transfers. Resistant lineages were obtained by supplementing MH with subinhibitory concentrations of amikacin and polymyxin B, which were increased 1.5-fold every four transfers, starting at one-fourth of the initial MIC values (amikacin: 1 μg·mL^–1^; polymyxin B: 0.5 μg·mL^–1^). This process resulted in three resistant lineages, designated as E2, E5, and E6.

### Resistance Stability Assay

To assess the stability of resistance, the three most resistant lineages from the in vitro evolution experiment (E2, E5, and E6) were monitored by comparing their MICs before and after 10 consecutive passages in antibiotics-free medium. Each lineage was first cultured in MH medium to a density of approximately 1 × 10^8^ CFU·mL^–1^, then transferred to a 96-well plate at ∼5 × 10^5^ CFU·mL^–1^ and incubated for 24 h at 37 °C with agitation at 80 rpm. Following incubation, cultures were passaged daily by transferring 1 μL of the culture to 100 μL of fresh MH broth. This procedure was repeated for 10 days, after which the MIC values were determined and compared with those obtained after the evolutionary trajectory.

## Fitness Cost Measurements

The bacterial lineages were cultured in MH medium and adjusted to ∼5 × 10^5^ CFU·mL^–1^. Aliquots of 100 μL were then dispensed into 96-well plates, and growth dynamics were monitored by measuring the optical density at 600 nm (OD600) at regular 30 min intervals under agitation, using Gen5 2.00 software. Seven independent assays were performed, each including the three resistant lineages (E2, E5, and E6), the parental lineage, the clinical isolate KP03, and the control lineage C1, which was propagated in the medium without antibiotics. Fitness parameters were then evaluated using the Automated Analysis of Microbial Growth Assays (AMiGA) software.^21^

### Cross-Resistance

The resistant lineage E6 and its parent strain, KP03, were tested for antimicrobial susceptibility using disk diffusion assays. Cross-resistance was defined as resistance to additional antimicrobials following the acquisition of resistance to amikacin and polymyxin observed in the E6 lines. Disk diffusion assays were carried out according to EUCAST standard procedures, and susceptibility or resistance was determined based on EUCAST zone diameter breakpoints. The antibiotics tested included ceftriaxone (30 μg), cefepime (30 μg), ertapenem (10 μg), Meropenem (10 μg), imipenem (10 μg), ciprofloxacin (5 μg), gentamicin (10 μg), and tigecycline (15 μg). Moreover, a microdilution assay was performed in MH medium supplemented with 2-fold diluted antimicrobial peptides (AMPs) to evaluate cross-resistance or susceptibility to colistin and to the AMPs. The final concentration of the tested bacterial strains was ∼5 × 10^5^ CFU·mL^–1^ in a final assay volume of 100 μL. The MIC was determined as the lowest concentration of AMPs that completely inhibited bacterial growth.

### Whole-Genome Sequencing Analysis

Genomic DNA was isolated from the parental lineage KP03, control lineages C1–C3, and resistant lineages E2, E5, and E6 using the Wizard Genomic DNA Purification Kit (Promega Corporation), according to the manufacturer’s protocol. Whole-genome sequencing was performed on the Illumina MiSeq platform, generating 150 bp paired-end reads with a sequencing depth exceeding 85x. Raw read quality was assessed with FastQCv.011.8 (https://www.bioinformatics.babraham.ac.uk/projects/fastqc/), and trimming was carried out with trimmomatic v.0.39 using the default parameters to remove adapters and low-quality bases.^22^ Trimmed reads of KP03 were assembled de novo with SPAdes v3.15.3. to generate a reference genome for analyzing mutations acquired during the evolutionary trajectory.^23^ Genome assembly was further assessed using Quast v5.0.2.^24^ Then, genome annotation was performed using Prokka v1.14.5.^25^ Mutation analysis, including single-base substitution (SNP), multiple-base substitution (SUB), insertions (INS), deletion (DEL), or sequence These resistant lineages exhibited lification (SA), which was conducted using Breseq v.035.2,^26^ with resistant and control strains compared against the parental lineage. Only the mutations presented in the resistant lineages but absent in the controls were considered in order to identify variants specifically associated with antibiotic resistance.

### RNA Extraction and Transcriptomics Analysis

For transcriptomics analysis, lineages E6 and C1 were first grown on agar plates following prior growth on an antibiotic-containing medium (amikacin and polymyxin combined). A preinoculum was prepared from a single colony of each lineage in 5 mL of MH medium and incubated at 37 °C and 200 rpm. Subsequently, an inoculum (1:100 dilution) was cultured under the same conditions until reaching ∼2 × 10 ^8^ CFU·mL^–1^, as determined by OD_600_, which was required for RNA extraction. Both lineages were then exposed for 1 h to half of their MIC values for amikacin and polymyxin. Total RNA was extracted for both lineages using an RNeasy Mini Kit (Qiagen) according to the manufacturer’s instructions. RNA quality was assessed using a Thermo Fisher Scientific NanoDrop 2000c spectrophotometer, and the concentrations were assessed by fluorimetry using a Qubit system (Invitrogen). RNA libraries were generated using Stranded Total RNA Prep with a Ribozero (Illumina) protocol and sequenced on the Illumina NextSeq 550 platform to generate 75 bp paired-end reads. Read quality was assessed using FastQC v.0.11.8, and alignment to the *K. pneumoniae* KP03 genome was performed using Bowtie2 (http://bowtie-bio.sourceforge.net/bowtie2/index.shtml). Gene counts were generated using HTseq^27^ and differential expression analysis was carried out using DESeq2.^28^ Differentially expressed genes were defined by a padj-value <0.01 and log2 fold-change ≥2 and ≤ −2 when comparing resistant and control lineages exposed to polymyxin B and amikacin.

Differential expression analysis of the *aphA* and *phoP* genes was assessed by comparing resistant lineage E6 with the control lineage C1 after exposure to antibiotics at 1/4 of the MIC for 1 h. Three biological and three technical replicates were analyzed using the 7300 Real-Time PCR System (Applied Biosystems, Foster City, CA, USA) with the SYBR green assay. Primer sequence for *aphA* (forward: TTTAGATCTTGGCCGTGCTG; reverse: TTAGCAGTTTCCTCCGATGC) and for *phoP* (forward: AGGATGGCTTCAGCGTACTG and reverse: TGCAGTTTATCCTGCTGCTG). Expression analyses were performed with real-time PCR Miner and REST (Relative Expression Software Tool) software.

Centro de Análises Proteômicas e Bioquímicas, Programa de Pós-Graduação em Ciências Genômicas e Biotecnologia, Universidade Católica de Brasília, Brasília71966–700, Brazil.

## Supplementary Material



## Data Availability

Genomic and transcriptomic sequences have been deposited with the Genebank databases under accessions PRJNA1100205 and GSE264574.

## Notes

Checkerboard assay results; antibiotic concentrations used during experimental evolution; MICs of amikacin and polymyxin B; resistance stability analyses; and growth parameter comparisons assessing fitness costs (PDF).
