# Supplementary material for: Rapid Adaptive Evolution under Combination Therapy in Klebsiella pneumoniae
Source: ACS Infect Dis. 2026 Feb 10;12(5):1627–36. doi: 10.1021/acsinfecdis.5c00934 (PMC13162261; doi:10.1021/acsinfecdis.5c00934)
Supplement: Supplementary file 1 [file id5c00934_si_001.pdf]

SUPPORTING INFORMATION

Title: Rapid Adaptive Evolution under Combination Therapy in *Klebsiella pneumoniae*

Camila Maurmann de Souza[1,2], Amy Lee[3], Osmel Fleitas Martínez[1], Kevin Ning[3], Mylena Cardoso da Costa[1], Mariana Rocha Maximiano[1,2], Gabriel Cidade Feitosa[1], Yasmim Neiva[1], Marcelo Campos[4], Marcelo Ramada[1,5], Sérgio Alencar[1], Robert E. W. Hancock[6], Octávio Luiz Franco*[1,2]

1 Centro de Análises Proteômicas e Bioquímicas, Programa de Pós-Graduação em Ciências Genômicas e Biotecnologia, Universidade Católica de Brasília, Brasília71966-700, Brazil

2 S-Inova Biotech, Programa de Pós-Graduação em Biotecnologia, Universidade Católica Dom Bosco, Campo Grande 79.117-900, Brazil

3 Department of Molecular Biology and Biochemistry, Simon Fraser University, South Sciences Building 7107, 8888 University Drive, Burnaby, British Columbia, Canada.

4 Integrative Plant Research Laboratory, Programa de Pós-Graduação em Biologia Vegetal, Universidade Federal de Mato Grosso, Cuiaba, 78060-900, Brazil.

5 Graduate Program in Gerontology, Catholic University of Brasilia, Brasilia-Brazil, Zip Code 71966-700

6 Centre for Microbial Diseases and Immunity Research, University of British Columbia, 2259 Lower Mall Research Station, Vancouver, British Columbia, Canada;

To whom correspondence should be addressed: Octávio L. Franco. Tel: 55 67 999854942

*Correspondence: ocfranco@gmail.com

Table S1: Checkerboard results of polymyxin B and amikacin combination against clinical isolated *Klebsiella pneumoniae* 03 (KP03). Synergism was defined as Fractional Inhibitory Concentration Index (FICI) FICI ≤ 0.5, indifferent/additive as 0.5 > FICI ≤ 4, antagonism FICI > 4.

Strain KP03	MIC alone (µg.mL^{-1})		MIC in combination (µg.mL^{-1})		
Replicates	Polymyxin B	Amikacin	Polymyxin B	Amikacin	FICI
1	2	4	2	4	2
2	1	2	1	2	2
3	2	4	2	4	2

Table S2: *Klebsiella pneumoniae* lineages used in this study. The last concentration of each antibiotic used to challenge the experimental lineages during evolutionary trajectory were also added. The parental and controls were not challenged with antibiotics (-). E2, E5, and E6 were capable to survive the highest concentration tested (">").

Klebsiella pneumoniae **Lineages**	**Feature**	**Last Survival concentration (µg.mL^{-1})**	
		**Polymyxin**	**Amikacin**
**KP03**	Clinical - carbapenemase (KPC)	-	-
**E1**	Experimental - challenged by antibiotic combination	19.2	38.4
**E2**	Experimental - challenged by antibiotic combination	> 43.2	> 86.5
**E3**	Experimental - challenged by antibiotic combination	3.84	7.64
**E4**	Experimental - challenged by antibiotic combination	43.2	86.5
**E5**	Experimental - challenged by antibiotic combination	> 43.2	> 86.5
**E6**	Experimental - challenged by antibiotic combination	> 43.2	> 86.5
**E7**	Experimental - challenged by antibiotic combination	28.8	57.7
**E8**	Experimental - challenged by antibiotic combination	43.2	86.5
**E9**	Experimental - challenged by antibiotic combination	43.2	86.5
**E10**	Experimental - challenged by antibiotic combination	43.2	86.5
**C1**	Control - without antibiotic challenge	-	-
**C2**	Control - without antibiotic challenge	-	-
C3	Control - without antibiotic challenge	-	-

Table S3: Minimal Inhibitory Concentration (MIC) of amikacin and polymyxin B against *Klebsiella pneumoniae* lineages after *in vitro* evolutionary trajectory. Experimental lineages submitted with amikacin and polymyxin B (E2, E5, E6) and control lineages without antibiotic submission (C1, C2, C3).

Klebsiella pneumoniae **lineages**	**MIC (µg.mL^{-1})**	
	**Amikacin**	**Polymyxin B**
**E2**	256	128
**E5**	128	128
**E6**	256	256
**C1**	2	2
**C2**	2	2
**C3**	4	2

Table S4: **Resistance stability of *Klebsiella pneumoniae* lineages after 10 days without amikacin and polymyxin B combination challenge.** Experimental lineages submitted with amikacin and polymyxin B (E2, E5, E6).

**Klebsiella pneumoniae linages**	**MIC (µg.mL^{-1})**			
	**Initial**		**After 10 days**	
	Amikacin	Polymyxin B	Amikacin	Polymyxin B
E2	256	128	256	128
E5	128	128	64	256
E6	256	256	256	256

Table S5: Comparison of growth parameters between *Klebsiella pneumoniae* resistant lineage E2 and control lineage C1.

**Parameter**	**Mean**		**95.0% CI**		**Sig. Dif.**
	**E2**	**C1**	**E2**	**C1**	
AUC (log)	118.565	123.308	[117.195,119.935]	[121.868,124.748]	Yes
Death (log)	0.522	0.653	[0.217,0.826]	[0.360,0.945]	No
Diauxie	0	0	NA	NA	No
Death Rate	-0.161	-0.165	[-0.327,0.005]	[-0.318,-0.011]	No
Growth Rate	1.261	1.413	[1.052,1.469]	[1.154,1.672]	No
Carrying Capacity (log)	5.698	6.088	[5.583,5.813]	[5.942,6.235]	Yes
Lag Time	0.104	0.239	[-0.149,0.358]	[0.056,0.421]	No
Adaptation Time	0	0	[0.000,0.000]	[0.000,0.000]	No
Time at Max. Death Rate	22.47	21.91	[18.460,26.480]	[17.389,26.431]	No
Time at Max. Growth Rate	0.19	0.07	[-0.517,0.897]	[-0.348,0.488]	No
Time at Carrying Capacity	12.79	15.23	[7.018,18.562]	[13.522,16.938]	No
Doubling Time	0.554	0.495	[0.463,0.645]	[0.401,0.589]	No

Table S6. Comparison of growth parameters between *Klebsiella pneumoniae* resistant lineage E5 and control lineage C1.

Parameter	Mean		95.0% CI		Sig. Diff.
	E5	C1	E5	C1	
AUC (log)	101.766	123.308	[99.667,103.864]	[121.868,124.748]	Yes
Death (log)	0.287	0.653	[-0.123,0.697]	[0.360,0.945]	No
Diauxie	0	0	NA	NA	No
Death Rate	-0.131	-0.165	[-0.342,0.079]	[-0.318,-0.011]	No
Growth Rate	0.834	1.413	[0.665,1.002]	[1.154,1.672]	Yes
Carrying Capacity (log)	5.226	6.088	[5.058,5.395]	[5.942,6.235]	Yes
Lag Time	0.322	0.239	[-0.078,0.723]	[0.056,0.421]	No
Adaptation Time	0	0	[0.000,0.000]	[0.000,0.000]	No
Time at Max. Death Rate	23.28	21.91	[19.873,26.687]	[17.389,26.431]	No
Time at Max. Growth Rate	0.435	0.07	[-0.694,1.564]	[-0.348,0.488]	No
Time at Carrying Capacity	19.92	15.23	[15.784,24.056]	[13.522,16.938]	No
Doubling Time	0.84	0.495	[0.670,1.010]	[0.401,0.589]	Yes

Table S7. Comparison of growth parameters between *Klebsiella pneumoniae* resistant lineage E6 and control lineage C1.

Isolate	Mean		95.0% CI		Sig. Diff.
	E6	C1	E6	C1	
AUC (log)	93.868	123.308	[91.708,96.028]	[121.868,124.748]	True
Death (log)	0.547	0.653	[0.109,0.985]	[0.360,0.945]	False
Diauxie	0	0	NA	NA	False
Death Rate	-0.183	-0.165	[-0.363,-0.004]	[-0.318,-0.011]	False
Growth Rate	0.793	1.413	[0.630,0.956]	[1.154,1.672]	True
Carrying Capacity (log)	4.819	6.088	[4.654,4.984]	[5.942,6.235]	True
Lag Time	0.448	0.239	[-0.027,0.923]	[0.056,0.421]	False
Adaptation Time	0	0	[0.000,0.000]	[0.000,0.000]	False
Time at Max. Death Rate	23.495	21.91	[20.728,26.262]	[17.389,26.431]	False
Time at Max. Growth Rate	0.765	0.07	[-0.764,2.294]	[-0.348,0.488]	False
Time at Carrying Capacity	14.755	15.23	[8.818,20.692]	[13.522,16.938]	False
Doubling Time	0.883	0.495	[0.701,1.066]	[0.401,0.589]	True
